# Near-Field Imaging of Hybrid Surface Plasmon-Phonon Polaritons on n-GaN Semiconductor

**DOI:** 10.3390/ma18122849

**Published:** 2025-06-17

**Authors:** Vytautas Janonis, Adrian Cernescu, Pawel Prystawko, Regimantas Januškevičius, Simonas Indrišiūnas, Irmantas Kašalynas

**Affiliations:** 1 Terahertz Photonics Laboratory, Center for Physical Sciences and Technology (FTMC), 10257 Vilnius, Lithuania; vytautas.janonis@ftmc.lt; 2 Attocube Systems AG, 85540 Haar, Germany; adrian.cernescu@attocube.com; 3 Laboratory of Semiconductor Characterization, Institute of High Pressure Physics PAS (UNIPRESS), 01-424 Warsaw, Poland; pprysta@unipress.waw.pl; 4 Ekspla Ltd., 02300 Vilnius, Lithuania; r.januskevicius@ekspla.com; 5 Laser Microfabrication Laboratory, Center for Physical Sciences and Technology (FTMC), 10257 Vilnius, Lithuania; simonas.indrisiunas@ftmc.lt

**Keywords:** gallium nitride (GaN), polar semiconductors, surface plasmon-phonon polariton, near-field imaging

## Abstract

Near-field imaging of the hybrid surface plasmon-phonon polaritons on the n-GaN semiconductor was performed using a scattering scanning near-field optical microscope at the selected frequencies of 920 cm^−1^ and 570 cm^−1^. The experimental measurements and numerical modeling data were in good agreement, revealing the large propagation distances on the n-GaN semiconductor and other insights which could be obtained by analyzing the dispersion characteristics of hybrid polaritons. In particular, the decay lengths of polaritons at the excitation frequency of 920 cm^−1^ were measured to be up to 25 and 30 µm in experiment and theory, respectively. In the case of excitation at the frequency of 570 cm^−1^, the surface plasmon-phonon polaritons’ decay distances were 25 µm and 105 µm, respectively, noting the limitations of the near-field optical microscope setups used. Dispersion characteristics of the resonant frequency and the damping rate of hybrid polaritons were numerically modeled and compared with the analytical calculations, validating the need for further experiment improvements. The launch conditions for the near-field observation of extraordinary coherence of the surface plasmon-phonon polaritons were also discussed.

## 1. Introduction

The field of modern nanophotonics is largely pushed forward by the ability to control light–matter interaction on a subwavelength scale, facilitated mainly by plasmonic structures in the range from visible to NIR wavelengths down to terahertz (THz) frequencies [1,2]. The modeling and material-level insights, particularly concerning field control, screening, and advanced dielectric response are crucial for the advancements in nanomaterial-based electromagnetic and quantum devices [3,4,5]. While surface plasmon polaritons (SPPs) provide new horizons in visual (VIS) and near-infrared (NIR) regions, a different approach is required in the range from THz to MID-IR due to relatively large plasmon losses [6]. In this regard, the surface phonon polaritons (SPhPs) come into play, taking advantage of the “metal–like” behavior of polar semiconductors in the Reststrahlen band region [7,8]. The phonon oscillations possess significantly smaller damping rates in crystal lattice than those of plasmons, leading to higher quality factors in exchange for a comparatively narrow spectrum range for operation [6]. The hybridization phenomenon of those two types of quasiparticles has been revealed recently via excitation of the surface plasmon-phonon polaritons (SPPhPs) in the relief grating of the heavily n-type doped GaN semiconductor [9]. Despite running debates on the propagation losses, the SPPhPs demonstrated directive and coherent thermal emission characteristics in the far-field. The quality factor of the SPPhP peak in the emission spectrum was measured to be small in comparison to the theoretical one, revealing limitations of the far-field experimental setup employing a detector with limited numerical aperture. In particular, the polariton dispersion possesses a so-called rainbow effect, where the emission frequency of the SPPhP depends on the observation angle. Thus, by using a detector with finite numerical aperture, the resonant feature appears to be wider in spectral measurements if the observation angle of the detector is large in comparison to the linewidth of the peak observed in the emission spectrum. Therefore, the direct probing of evanescent wave fields and a more rigorous theoretical description of the microscopic SPPhP excitation are required when expecting to expand the practical applications of hybrid polaritons. Development of the high-coherence polaritonic systems is highly important for various applications including sensing [1], energy conversion and emission [10,11], radiative cooling [12,13], and even quantum technologies [4].

The imaging of evanescent electromagnetic fields, including those of surface polaritons, became available recently by the advancement of scattering scanning near-field optical microscope (s-SNOM) technology [2]. New tolls provide insight into the physics and dynamics of the resonant surface polariton features, which were successfully imaged in various structures based on semiconductors, metals, and 2D Van der Waals materials [14] in the range from THz [5] to NIR [15] and visible [16]. In particular, the s-SNOM in the spectrum range of 890–930 cm^−1^ has been used for direct imaging of the SPhPs launched at the Au edge deposited on SiC crystal, where a data quantitative analysis revealed large distances of phonon polaritons’ propagation reaching values up to 200 μm [8]. Recent advancements and research on plasmon–phonon hybridization mainly focus on plasmon–phonon interaction in layered 2D materials [17,18], while the SPPhPs in a homogenous doped semiconductor structure remain comparatively new and unexplored, with work ongoing even at the theoretical level [19].

In this work, the hybrid SPPhPs were launched at room temperature on the n-GaN semiconductor, near the edge of surface relief grating, using a commercial s-SNOM at two selected frequencies of 570 cm^−1^ and 920 cm^−1^. The wavevector matching conditions were preserved for the counter-propagating (negative) and co-propagating (positive) modes by changing the incidence direction of the laser beam with respect to the grating edge. The usage of focused and defocused laser beams was also discussed measuring the short- and long-range polariton propagation within a distance range of up to 100 µm. The experimental data were modeled numerically employing the Finite-Difference Time-Domain (FDTD) methods implemented in CST Studio. The decay lengths of polaritons at the 920 cm^−1^ excitation frequency were 25 µm and 30 µm in experiment and theory, respectively. Meanwhile at the excitation frequency of 570 cm^−1^, the decay lengths were measured to be up to 25 µm and 105 µm in experiment and theory, respectively. The difference in decay length values of hybrid SPPhPs for the latter case was attributed to experimental setup limitations, illustrating the need for the usage of separated excitation and probing schemes. While the SPPhP damping rates were constrained in the experiment setup, the dispersion of numerical and experimental frequency values was found in good agreement. Moreover, the numerical calculations of the damping rate dispersion showed good agreement with the analytical model, validating the approach employed. Finally, we discussed the possible experimental launch and near-field mapping of the hybrid polaritons at the frequencies, which matching the transverse optical phonon frequency and the lattice wavevector of surface relief grating. This work marks successful near-field imaging of the hybrid SPPhPs launched on the n-GaN semiconductor with the first direct comparison of amplitudes between SNOM and FDTD data.

## 2. Materials and Methods

A 10 × 10 mm^2^ size sample was fabricated out of a bulk n-GaN semiconductor possessing a free charge carrier density of 1.5 × 10^19^ cm^−3^. The high concentration of free electrons allowed the widening of the Reststrahlen band. The LO phonon mode from 734 cm^−1^ for the undoped GaN was transformed into the coupled plasmon—LO phonon mode found at the 1140 cm^−1^ frequency for doped GaN. A surface relief grating of the n-GaN semiconductor was optimized with the ridge height, periodicity, and filling factor set to values of 1 µm, 17.5 µm, and 25%, respectively.

The peculiarities of the SPPhP excitation and propagation from the edge of the periodic grating were numerically investigated by performing FDTD calculations (CST Studio 2024, Dassault Systèmes Simulia, Providence, Rhode Island, USA). The modeled structure was 350 μm wide (*y*-axis) and 3350 μm long (*x*-axis) and had 20 grating features with other grating parameters the same as in the design of the fabricated sample. The mesh size of around 45 million cells was used with solver accuracy set at −40 dB. Open boundary conditions were selected with the distance from the modeled structure set at lambda/4 at the maximum modeled frequency and an estimated reflection level of 1 × 10^−4^. In total, 200 µm of open space was added above the grating structure with the plane wave excitation inbound at set incidence angles. A linearly polarized, transverse magnetic (TM) plane wave with the electric field amplitude of 1 V/m was used. The line profiles of the electric field amplitude were extracted from the flat open area of the modeled structure. The electrical field amplitude was monitored in the *x*-axis direction, in the center of the modeled structure, and in the y-direction, at the height over the surface of z = 0.5 µm.

A description of the dielectric function of the material was needed for numerical modeling. The dielectric function of n-GaN was defined via the Lorentz–Drude function given in Equation (1), the parameters of which are summarized in Table 1 [9].

(1) $$\varepsilon =\varepsilon_{\infty }\left(\frac{\nu_{LO}^{2}-\nu^{2}-i\nu \Gamma_{LO}}{\nu_{TO}^{2}-\nu^{2}-i\nu \Gamma_{TO}}-\frac{\nu_{p}^{2}}{\nu^{2}+i\nu \Gamma_{p}}\right),$$

A commercial s-SNOM (attocube systems AG, Munich, Germany) operating in a pseudo-heterodyne regime was equipped with the Quantum Cascade Laser (QCL) and Optical Parametric Oscillator (OPO) based on the Ekspla laser used at the frequencies of 920 cm^−1^ and 570 cm^−1^, respectively. The sample was scanned in a large area, reaching width and length values up to 100 µm and 20 µm, respectively. The topography map (Z), scattered optical field amplitude (A), and phase (P) of pseudo-heterodyne signals were simultaneously recorded at each scanning point of the sample.

**Figure 1:**
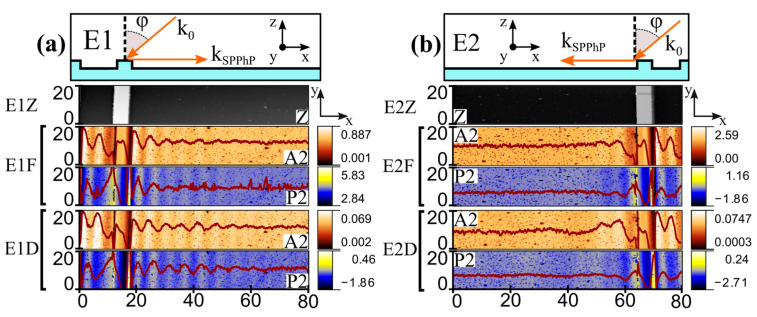
The s-SNOM images at excitation frequency of 920 cm^−1^ for the experiment geometry E1 (**a**) and E2 (**b**), illustrated in top insets. Scans were performed over 80 µm length (x) and 20 µm width (y) area. Topography plot of scanned sample area (Z), contour plot of the second harmonic amplitude (A2), and phase (P2) were obtained using focused (E1F, E2F) and defocused (E1D, E2D) laser beams. Solid line superimposed in every contour plot was found by averaging 45 scan lines for a respective measurement.

During the SNOM experiment, the laser illumination at the incident angle *φ* excited the hybrid polariton with the wavevector, *k*_SPPhP_, in parallel with the grating vector. The excitation of SPPhP mode with either a “negative” (E1 geometry) or “positive” (E2 geometry) sign can be carried out by rotation of the grating by 180 degrees around the *z*-axis. The schematic illustrations of experimental geometries E1 and E2 are shown in the insets of Figure 1a,b, respectively. The SPPhP oscillations were experimentally recorded on the flat side of the n-GaN surface, propagating in a normal direction away from the grating edge.

The launch of hybrid SPPhPs from the AFM tip is not efficient because the small diameter tip can only launch polaritons with high wavevectors (*k*_TIP_ ≈ 1/*d*_TIP_ ≈ 1/20 nm^−1^) [20,21]. Therefore, we stated that only grating edge-launched polaritons were involved in our experiments.

In most s-SNOM setups, a laser beam is tightly focused on the AFM tip exciting and probing the evanescent electromagnetic fields in close vicinity of the tip [15]. In this case, the laser beam is centered on the AFM tip and focused as much as possible to maximize the signal-to-noise ratio. However, in our work, the laser spot diameter should be maximized to probe the grating-launched SPPhPs as far from the grating edge as possible. Thus, we explored two polariton excitation and probing regimes. In the first case, the laser beam was focused on the AFM tip providing a maximum s-SNOM signal, but a relatively small illumination area. In another case, the laser beam was defocused to increase an effective scan area; those polaritons were launched on n-GaN crystal when the grating edge was kept illuminated during the entire scan, as described elsewhere [8]. The s-SNOM signal level decreased by approximately 10 times; however, the observed signals of SPPhP oscillations became more pronounced.

The second SNOM demodulation harmonic was selected for the analysis of the experimental data. The first harmonic of the SNOM signal demodulation was not analyzed due to its intense interference with excitation light [22]. The third and fourth demodulation harmonics were not analyzed due to very similar dependencies when compared to the second harmonic, but with smaller s-SNOM signal levels and signal-to-noise ratios.

## 3. Results

The measured SNOM topography (z), amplitude (A), and phase maps for the experiments at the excitation frequency of 920 cm^−1^ and in E1 and E2 configurations are shown in Figure 1a,b, respectively. In the topography maps, the last grating feature was observed, coinciding in the x position with the schematic representation in the top insets of Figure 1. The s-SNOM amplitude and phase traces demonstrated very similar oscillating behavior (i.e., a similar number of oscillatory features present with similar decay). Superimposed on the amplitude and phase maps, the signal curves obtained by averaging all the adjacent measurement lines are also shown. The observed fringe spacings between the signal amplitude oscillations were found to be the same for the focused (F) and defocused (D) regimes. Experiments were carried out by positioning the sample in E1 and E2 geometries, showing different SPPhP fringe spacing (periodicities) in different experimental configurations.

**Figure 2:**
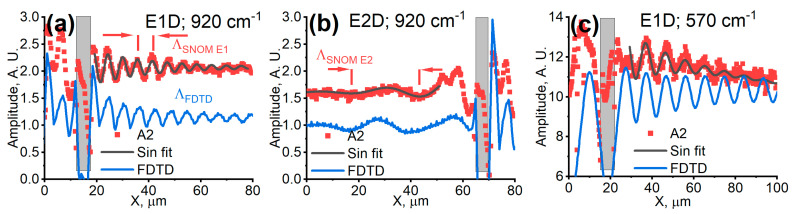
Comparison between the s-SNOM-measured (red dots) and FDTD-modeled (blue lines) signals versus the surface x-coordinate X at the excitation frequency of 920 cm^−1^ in the geometry E1D (**a**) and E2D (**b**), and at 570 cm^−1^ in the geometry E1D (**c**). The SNOM-measured signal is normalized and offset to directly compare the measured and simulated traces. An exponentially decaying sinusoid fitted to measurement results is shown (gray lines) and used for extraction of the decaying distances.

Experimental amplitude data were fitted to a single-frequency sinusoid exponential decay model. The results are shown in Figure 2. At the frequency of 920 cm^−1^ and E1 geometry, the fringe spacing and exponential decay distance from A2 (second SNOM demodulation harmonic) characteristics were found to be $\Lambda_{E1}=6$ μm and 25 µm, respectively.

In the case of geometry E2, the fringe spacing of SPPhP oscillations was found to be larger when compared to geometry E1. Signals were more pronounced in the case of defocused laser beam (E2D) usage rather than focused (E2F), despite a notably lower overall s-SNOM signal level (see Figure 1b). The long-range oscillations were observed lasting around one period with fringe spacing of $\Lambda_{E2}=26$ μm before apparent complete decay. The exponential decay distance was measured to be up to 20 µm. The short-range oscillations with the period of about 6 µm lasting for one and a half cycles were observed near the grating ridge. These were the evidence of the excitation of leaky evanescent waves in the vicinity of the grating.

When carrying out SNOM experiments, the angle of the incoming beam onto the sample may deviate from the initial value provided in the instrument documentation (60 degree) due to the defocusing procedure. However, for result interpretation and modeling, it is essential to know the incidence angle, which is specific for each setup. Fortunately, the SPPhP fringe spacings, *Λ*, obtained in experiments E1 and E2, allowed us to evaluate the incidence angle of the electromagnetic wave. The equation for matching wavevector momenta for the laser light and the scattering field feature resulted in the incident angle of [21,23]:

(2) $$\varphi =\sin^{-1}\left(\frac{\pi }{k_{0}}\left(\frac{1}{\Lambda_{E1}}-\frac{1}{\Lambda_{E2}}\right)\right),$$

where $\Lambda_{E1}$ and $\Lambda_{E2}$ are the periods of SPPhP oscillations propagating in backward and forward directions, respectively. The measured respective periods from SNOM experiments (see Figure 2) were used to find the incidence angle in the experiment at 920 cm^−1^. The value was found to be at about 44 degrees, which was different from the value indicated by the instrument producer at the nominal operation using the tightly focused laser beam.

The modeling results in direct comparison to the experimental measurements are shown in Figure 2. The modeled electric field distributions at the excitation laser frequency of 920 cm^−1^, for geometries E1 and E2, are shown in Figure 2a,b, respectively. The oscillations of electric field amplitude began with the ridge of the grating. A good match was achieved between the experimental and modeling data. The modeled traces in the flat sample region revealed a long-range SPPhP propagation with the decay distance of the oscillations about 28 µm and 30 µm for the geometries E1 and E2, respectively. These values also coincided well with the SPPhP coherence length values, obtained using numerical calculations by the Rigorous Coupled Wave Analysis (RCWA) method when investigating far-field radiation directivity profiles at this frequency [9].

The results obtained at the excitation frequency of 570 cm^−1^ are shown in Figure 2c. In this case, the modeled results demonstrated a considerably longer oscillation distance where SPPhPs oscillated with a fringe spacing of about 9 μm. The decay distance was found from the FDTD results, revealing values of about 105 μm, while those from experimental data were only about 20 μm. The discrepancy was attributed to the finite diameter of the excitation laser spot. Although the excitation beam spot diameter was not directly measured, we estimated it to be of a similar value to the measured decay distance, i.e., about 30 μm, limiting the measurements of large decay distances of hybrid SPPhPs [24].

Moreover, at the frequency of 570 cm^−1^, the periodicity of observed oscillations revealed that the incidence angle in the experiment was around 60 degrees, the value of which differed from that obtained for experiments at 920 cm^−1^. The change in the value was attributed to the realignment of the setup with the change of the excitation laser. Considering that the measurement of incidence angles within an individual s-SNOM system is shown not to be a trivial task, the excitation and monitoring of oscillation traces of the surface polaritons can help to adjust and calibrate the experimental setup.

**Figure 3:**
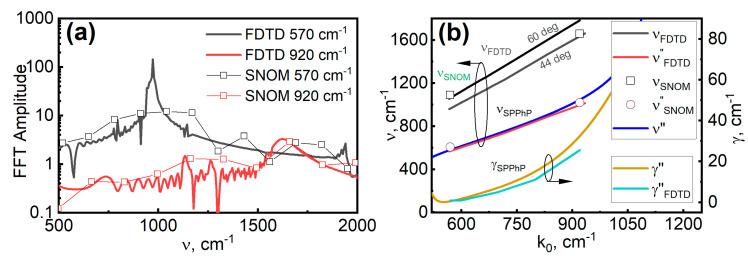
(**a**) FTT spectra of measured and modeled SPPhP excitations at frequencies of 570 cm^−1^ and 920 cm^−1^. (**b**) The polariton modeled ν_FDTD_ and measured ν_SNOM_ apparent wavevectors, the respective $\nu^{\prime \prime }$_FDTD_ and $\nu^{\prime \prime }$_SNOM_, genuine frequencies with modeled γ_SPPhP_ damping rate in comparison to the polariton frequency ν″ and damping rate γ″ obtained from analytical calculations.

Next, we investigated the dispersion of the hybrid SPPhP frequency and damping rate when launched on the n-GaN semiconductor. For this, the oscillation spectra of s-SNOM and FDTD signals, related to polariton propagation, were found performing Fast Fourier Transform (FFT) of measured and simulated amplitude signals in the flat region of n-GaN. The results are shown in Figure 3a. This study marks the first comparison of absolute amplitudes between SNOM and FDTD data.

The overall tendency was similar between the measured and simulated data at both 920 and 570 cm^−1^ frequencies. However, the resolution of the experimental FFT results was notably lower due to the experimental scanning distance of around 100 µm being considerably smaller than that used for modeling (around 3 mm). As a result, the sharp feature at a frequency of 570 cm^−1^ was not distinguished in the experiment. Nevertheless, at the frequency of 920 cm^−1^, the modeled and experimental FFT data demonstrated good agreement despite small distances employed in imaging. Therefore, we can conclude that usage of a different s-SNOM setup is required for the investigation of hybrid SPPhP decay length at the excitation frequency of 570 cm^−1^, i.e., the spectrum region where the transverse optical phonon frequency and the lattice wavevector of surface relief grating are comparable in size.

The numerical modeling was performed at other excitation frequencies and two incident angles found from the experiments. The peak frequency ($\nu_{A}$) and linewidth (*W*) parameters were extracted by fitting the Pearson VII function (Origin library) to the modeled FFT spectra. The polariton damping was calculated as *γ*_FFT_ = *W*/2π. The results are shown in Figure 3b given by the black color line. We see that the SPPhP frequencies extracted from the traces (*ν*_SNOM_, *ν*_FDTD_) were higher than the one predicted by the analytic dispersion curve given by the solid blue line. This was due to the self-interference of SPPhP oscillations with incoming radiation increasing the apparent polariton frequency. The genuine polariton wavevector ν_SPPhP_ that is launched on the grating edge can be found from the apparent spatial frequency $\nu_{A}$, using the equation [25]:

(3) $$\nu_{SPPhP}=\nu_{A}-k_{0}\sin(\varphi ),$$

where *ν*_A_ = *ν*_SNOM_= 1/*Λ*_SNOM E1_ in the case of s-SNOM measurement; *ν*_A_ = *ν*_FDTD_= 1/Λ_FDTD_ (or the maximum frequency of the FFT) in the case of FDTD modeling. The values of the genuine SPPhP wavevector recalculated from the FDTD data were close to the values of the analytical SPPhP frequency dispersion line reported in the literature [9]. The genuine wavevector values obtained from the s-SNOM data also followed the analytical dispersion characteristic quite closely when we considered the incidence angle values of 60 and 44 degrees for experiments at 570 cm^−1^ and 920 cm^−1^, respectively. Moreover, the dependency of the modeled polariton damping rate closely followed the analytical dispersion characteristic in the whole spectrum range. The observed divergence of the extracted damping results of around 20 percent from the analytical curve was attributed to the extraction procedure, where parameter *W* did not directly translate to the FWHM value of the asymmetrical, complex form of the observed FFT peak. Overall, these results demonstrate that the s-SNOM measurements and the FDTD modeling allowed near-field imaging of hybrid polaritons launched on the n-GaN surface. However, a quantitative analysis of the polariton decay length (travel distance and damping rate) from experimental data requires some further optimization steps.

For example, even higher field amplitudes and travel distances (lower damping rates which translate into larger coherence length values) have been observed by RCWA calculations for the fundamental hybrid SPPhP mode, excited at the 570 cm^−1^ frequency, at a normal incidence angle [24]. The quality factor was found to be up to *Q* = 570. In this spectrum region, a fundamental mode possessed a spectral linewidth of about 1 cm^−1^ with the spatial coherence length of up to 9 mm, thus providing extremely large propagation distances of the hybrid polaritons on the n-GaN surface [9,24]. However, a near-field investigation of such excitations requires another experimental approach, allowing one to launch the polaritons at a normal incidence angle and to investigate the polariton traveling at distances much larger than the laser beam spots, available in the currently used s-SNOM system. One such example could be a back–side illumination through a thin GaN film, or, alternatively, a decoupled near-field launch and probe schemes, as were recently demonstrated using an automated double-tip SNOM system [26].

## 4. Conclusions

We have demonstrated experimentally the near-field imaging of the hybrid plasmon-phonon polaritons on the surface of the n-GaN semiconductor launched near the edge of surface relief grating. Long-range SPPhP oscillations were observed at room temperature at two laser frequencies of 920 cm^−1^ and 570 cm^−1^. The decay distance values up to 25 µm (105 µm) in the experiment (theory) were observed at a frequency of 570 cm^−1^. Numerical finite difference time domain modeling closely followed the experimental measurement results at a frequency of 920 cm^−1^. The discrepancy between the experiments and modeling at 570 cm^−1^ revealed the need for decoupling of the excitation and probing laser beams in the s-SNOM system to obtain larger distances in the experiment. Meanwhile, the numerical calculations of the damping rate dispersion showed good agreement with the analytical model, validating the implemented approach and demonstrating, however, that damping rate values of hybrid SPPhPs were constrained in our experiments. We also confirmed that measured periods in traces of forward- and backward-direction propagating hybrid polaritons can be used to find the incidence angle of the laser beam in SNOM setups. Finally, this work marks the first direct comparison of amplitudes between SNOM and FDTD data of near-field SPPhP oscillations obtained on the surface of the n-GaN semiconductor.

## Figures and Tables

**Table 1:** Parameters of dielectric function of used n-GaN semiconductor.

Parameter	ν_TO_ (Γ_TO_) [cm^−1^]	ν_LO_ (Γ_LO_) [cm^−1^]	υ_p_ (Γ_p_) [cm^−1^]	ε_∞_
Value	557 (7)	739 (7)	1145 (390)	5.3

## Data Availability

The original contributions presented in this study are included in the article. Further inquiries can be directed to the corresponding author.
